# The impact of urine collection method on canine urinary microbiota detection: a cross-sectional study

**DOI:** 10.1186/s12866-023-02815-y

**Published:** 2023-04-13

**Authors:** Emily L. Coffey, Andres M. Gomez, Aaron C. Ericsson, Erin N. Burton, Jennifer L. Granick, Jody P. Lulich, Eva Furrow

**Affiliations:** 1 University of Minnesota, 1352 Boyd Avenue C339 Veterinary Medical Center, 55108 Saint Paul, MN USA; 2 University of Missouri, 4011 Discovery Drive S123B, 65201 Columbia, MO USA

**Keywords:** Urinary microbiota, Canine, Urine collection technique, Cystocentesis, Midstream voiding

## Abstract

**Background:**

The urinary tract harbors unique microbial communities that play important roles in urogenital health and disease. Dogs naturally suffer from several of the same urological disorders as humans (e.g., urinary tract infections, neoplasia, urolithiasis) and represent a valuable translational model for studying the role of urinary microbiota in various disease states. Urine collection technique represents a critical component of urinary microbiota research study design. However, the impact of collection method on the characterization of the canine urinary microbiota remains unknown. Therefore, the objective of this study was to determine whether urine collection technique alters the microbial populations detected in canine urine samples. Urine was collected from asymptomatic dogs by both cystocentesis and midstream voiding. Microbial DNA was isolated from each sample and submitted for amplicon sequencing of the V4 region of the bacterial 16 S rRNA gene, followed by analyses to compare microbial diversity and composition between urine collection techniques.

**Results:**

Samples collected via midstream voiding exhibited significantly higher sequence read counts (*P* = .036) and observed richness (*P =* .0024) than cystocentesis urine. Bray Curtis and Unweighted UniFrac measures of beta diversity showed distinct differences in microbial composition by collection method (*P =* .0050, R^2^ = 0.06 and *P =* .010, R^2^ = 0.07, respectively). Seven taxa were identified as differentially abundant between groups. Pasteurellaceae, *Haemophilus, Friedmanniella*, two variants of *Streptococcus*, and *Fusobacterium* were over-represented in voided urine, while a greater abundance of *Burkholderia-Caballeronia-Paraburkholderia* characterized cystocentesis samples. Analyses were performed at five thresholds for minimum sequence depth and using three data normalization strategies to validate results; patterns of alpha and beta diversity remained consistent regardless of minimum read count requirements or normalization method.

**Conclusion:**

Microbial composition differs in canine urine samples collected via cystocentesis as compared to those collected via midstream voiding. Future researchers should select a single urine collection method based on the biological question of interest when designing canine urinary microbiota studies. Additionally, the authors suggest caution when interpreting results across studies that did not utilize identical urine collection methods.

**Supplementary Information:**

The online version contains supplementary material available at 10.1186/s12866-023-02815-y.

## Background

Emerging research has established that the urinary tract, previously thought to be sterile, hosts a unique and diverse microbial ecosystem [1–6]. Urinary microbial communities play essential roles in urogenital health and disease, and disorders such as urinary incontinence, urolithiasis, and bladder neoplasia have been linked to alterations in human urinary microbiota (UM) [6–12]. Further research investigating the UM is critical to improving our understanding of host-microbe relationships within the urinary tract and exposing novel treatment and preventative strategies for disorders associated with urinary dysbiosis.

Microbiota analyses of the urinary tract present several unique challenges for researchers. Most notably, urine possesses low biomass, which creates vulnerability to contamination and technical biases. To address these challenges, UM studies require careful specimen collection, handling, and bioinformatic processing. Sample collection technique represents a particularly critical component of UM study design, as biological sources of contamination vary by urine collection method. Urine samples are typically collected by either midstream voiding (i.e., free catch), urethral catheterization, suprapubic/antepubic aspiration (i.e., cystocentesis), or cystoscope evacuation. In humans, sample collection technique alters UM composition [1, 10, 13–17]. Voided samples risk contamination with urethral, genital, and dermal microbiota, whereas cystocentesis and urethral catheterization are thought to better reflect the microbiota of bladder urine. In fact, a recent consensus statement recommends differentiating urinary tract microbiota terminology, reserving the designation “urinary bladder” for samples collected directly from the bladder (urethral catheterization, cystocentesis, or cystoscopic collection), and using the term “urogenital” for voided samples [12].

The dog is a valuable model for urogenital and microbiota-related research, and investigation of the canine UM might inform disease pathology relevant to both canine and human health, such as urolithiasis, urinary tract infections, neoplasia, and neurogenic bladder [18–21]. The presence of urinary microbial communities in dogs is also well-established [22–26], yet the impact of urine collection method on microbiota composition in dogs remains unknown. Past studies examining the canine UM have included urine collected by either cystocentesis or midstream voiding, which are two of the most common methods of canine urine collection performed in clinical settings [22–26]. The microbiota of canine urine collected via cystocentesis shares more similarities with the microbiota of genital swabs than rectal swabs from the same animal [22]. However, the effect of urine collection technique on canine UM detection remains unknown. This is a critical issue given that urethral, genital, or dermal microbial contamination of voided urine might alter UM composition in dogs.

Improved understanding of how urine sample collection affects these microbial communities is essential for the design and interpretation of canine UM studies. Therefore, the primary objective of this study was to characterize the impact of urine collection method on microbial abundance, diversity, and composition in canine urine samples. A secondary objective was to determine how specific aspects of the analysis pipeline, including minimum read count (i.e., sequencing depth) and data normalization method, affect results.

## Methods

### Patient selection

Spayed or neutered dogs between the ages of 1 and 7 years old were actively recruited through an email announcement about the study to employees at the University of Minnesota’s College of Veterinary Medicine until a total of 10 male and 10 female dogs meeting the study inclusion criteria were enrolled. Dogs with a diagnosis of lower urinary tract disease or clinical signs localizing to the lower urinary tract were excluded. Clinical signs of lower urinary tract disease were defined as gross hematuria, stranguria, pollakiuria, or dysuria. Dogs with active dermatologic disease or visible dermatologic lesions in the perivulvar, preputial, or inguinal areas were not included. Dogs with a diagnosis or clinical signs of chronic or acute (within 3 weeks of study enrollment) gastrointestinal disease were also excluded. Clinical signs of gastrointestinal distress included vomiting, diarrhea, hematochezia, borborygmus, hyporexia, anorexia, or abdominal bloating. Finally, dogs that received antimicrobial or immunosuppressive therapies within 3 months of study enrollment were excluded. Signalment, body weight, body condition score (BCS, 1–9 scale), hair length, presence of dermatologic lesions, medical history, diet, medication history, urinalysis results, and urine culture results were recorded for each study participant. Hair length was categorized as short (< 1 inch), medium (1–2 inches), or long (> 2 inches).

### Sample collection

For each participant, antepubic cystocentesis was performed first, followed by collection of voided urine. Skin was cleansed with 70% isopropyl alcohol prior to cystocentesis. Five mL of urine was collected via cystocentesis, and the collection needle was exchanged for a sterile needle prior to transfer to a sterile tube. A minimum of 7 mL of midstream, voided urine was collected in a sterile cup by a veterinarian or certified veterinary technician within 6 h of cystocentesis. Samples were refrigerated immediately after collection and transported to a -80˚ C freezer within 2 h of collection. The time between cystocentesis and voided urine collection was documented for each dog. If a dog voided immediately after cystocentesis, the interval was recorded as 0.1 h.

An aliquot of the voided urine sample ranging from 1 to 6 mL was submitted for urinalysis. Dogs with pyuria (> 5 white blood cells/hpf) or cytologic bacteriuria were excluded. Urine specific gravity (USG) was measured using a digital veterinary refractometer (MISCO Palm Abbe, Solon, OH), and urine pH was determined using a urine dipstick chemistry test (Siemens MultiStix, Malvern, PA). For dogs with a USG reported as > 1.045, the USG was recorded as 1.045 for analyses. Aerobic bacterial culture was also performed from voided urine from each dog. Approximately 0.5 mL of urine was gently mixed, and a 1 µL inoculation loop (Globe Scientific, Mahwah, New Jersey) was used to perform a streak plate inoculation of a Blood Agar Plate (Hardy Diagnostics, Santa Maria, California), followed by incubation at 37˚ C for 48 h. Significant bacterial growth was defined as greater than or equal to 10^5^ CFU/mL according to previous guidelines [27]. Thus, samples with bacterial growth ≥ 10^5^ CFU/mL after 48 h of incubation were excluded.

### DNA isolation and amplicon sequencing

Five mL of urine from each collection method were reserved for bacterial 16 S rRNA gene amplification and sequencing. Prior to DNA extraction, urine was pelleted by centrifugation at 3,000 rpm for 15 min [28]. The DNeasy PowerSoil Pro Kit (QIAGEN, Hilden, Germany) was used to extract microbial DNA from the urine in accordance with manufacturer instructions. Two negative controls were included, which consisted only of DNA extraction reagents. The ZymoBIOMICS Microbial Community Standard (Zymo Research, Irvine, California) was used as a positive control. This is a mock bacterial community comprised of eight organisms: *Pseudomonas aeruginosa, Escherichia coli, Salmonella enterica, Lactobacillus fermentum, Enterococcus faecalis, Staphylococcus aureus, Listeria monocytogenes*, and *Bacillus subtilis.*

DNA concentration and purity was determined using Quanti-iT PicoGreen dsDNA Assay kit (Invitrogen, Waltham, MA) and NanoDrop spectrophotometry (Thermo Fisher Scientific, Waltham, MA). The V4 region of the bacterial 16 S rRNA gene was amplified using primers 515 F (GTGCCAGCMGCCGCGGTAA) and 806R (GGACTACHVGGGTWTCTAAT) [29]. Amplicon sequencing of all clinical samples, negative controls, and the positive control were performed on the MiSeq sequencing platform, targeting 2 × 300 bp paired-end reads, v3 chemistry (Illumina, San Diego, CA). DNA extractions and library preparation were performed in a single batch. DNA extraction, library preparation, PCR amplification, and 16 S rRNA short amplicon sequencing were performed at the University of Minnesota Genomics Center.

### Bioinformatic processing

Raw, paired-end sequence reads were processed using Cutadapt [30] for primer removal, followed by QIIME2 (v. 2020.8) [31] and DADA2 [32] for the removal of low-quality base pairs, sequence truncation, and forward and reverse read merger. Taxonomic assignment of high-resolution amplicon sequence variant (ASV) outputs was performed using the Silva reference database (v. 138) [33]. Chimeras, mitochondria, chloroplasts, ASVs with unassigned taxonomy, and ASVs present in only a single sample were removed from the dataset prior to downstream analysis. Sequences represented in less than 10 total reads across all samples were also removed, as previously described [8, 34]. The *decontam* package (v. 1.10.0) within the R statistical software was used to identify contaminant taxa using the prevalence method with a threshold of 0.5 [35]. Features identified as putative contaminants via *decontam* were removed from the dataset prior to downstream analysis. A threshold of 300 sequence reads was set for the primary analyses, and any sample with less than 300 reads was removed from analysis [24]. Only data from dogs with paired cystocentesis and voided urine samples meeting these criteria were included in analyses. Sequence read counts were normalized via relative abundance transformation for the primary analyses.

### Data analysis

Normality of continuous variables was determined using the Shapiro-Wilk test within the R statistical software (v. 4.0.2). The *vegan* (v. 2.5-7) [36] and *phyloseq* (v. 1.34.0) [37] packages were used to perform alpha and beta diversity analyses. Alpha diversity measures included Shannon diversity index, inverse Simpson diversity index, observed richness, and Pielou’s evenness. Alpha diversity was compared between experimental groups using Wilcoxon signed-rank test (analysis by collection method) or Wilcoxon rank-sum tests (analysis by sex). Measures of beta diversity included Bray Curtis dissimilarity, Weighted UniFrac, and Unweighted UniFrac. Statistical differences in microbial composition between groups (urine collection method and sex) was assessed using permutational multivariate analysis of variance (PERMANOVA) with 1000 permutations. A two-way PERMANOVA (adonis2 function in R *vegan* package) was also performed to assess for interactions between urine collection method and sex.

Identification of differentially abundant organisms between experimental groups was performed using indicator species analysis [38] in the R *labdsv* package (v.2.0–1) [39]. Indicator species analysis assigns an indicator value (IV) to each ASV by calculating the product of the relative frequency and relative abundance of an individual taxon in an experimental group [38]. IVs range from 0 to 1, with higher values indicating that a taxon has higher abundance within samples and is more frequent across samples of a given experimental group. To be considered a significant differential feature by indicator species analysis, an IV of > 0.3 and a *P* value of < 0.05 was required, as previously described [40]. Indicator species analysis was performed between collection method, sex, and between sexes within a given collection method group. Organisms identified as differentially abundant by indicator species analysis were also evaluated with Wilcoxon signed-rank test (analysis by collection method) or Wilcoxon rank-sum test (analysis by sex) to compare overall abundance of organisms between experimental groups. Random forest analysis (classification mode; *randomForest* package v. 4.6–14) was also performed, and features with the highest discriminatory power between collection method groups using the mean decrease gini index were identified.

These data were further investigated using four additional sequence read thresholds (100, 700, 1000, 2000) and three normalization methods (performed at each sequence read threshold, including the primary threshold of 300). Normalization methods included rarefaction with random subsampling to the designated sequence read threshold (rrarefy function in R *vegan* package), relative abundance transformation, and negative binomial distribution using DESeq2 (v. 1.30.1) [41, 42]. A rarefaction curve was created (rarecurve function in R *vegan* package) to assess whether maximum diversity was achieved at the various sequence read thresholds.

## Results

### Study participants

**Fig. 1:**
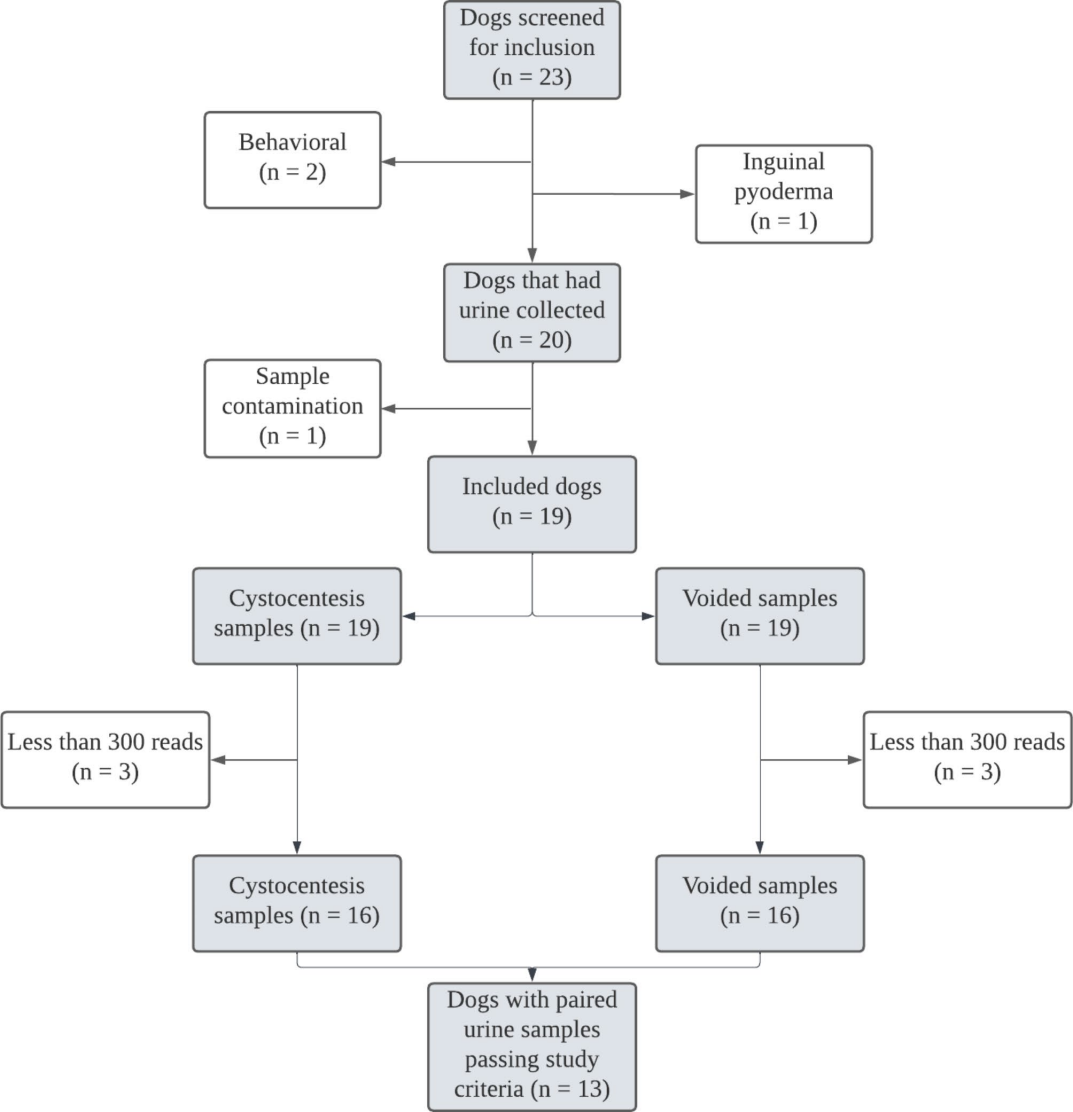
Flow diagram showing the indications for exclusion of dogs and urine samples

Twenty-three dogs were recruited and screened for inclusion. Three dogs were excluded from the study during the sample collection phase. Two were excluded for behavioral causes, as the dogs’ temperaments prohibited urine collection, and the third was excluded due to visible inguinal pyoderma at the time of sample collection. Of the 20 dogs with urine samples collected, one female dog was later excluded due to contamination of her samples with the positive control during sample processing. The final study group comprised 19 dogs, including 10 neutered males and 9 spayed females. Indications for participant exclusions are summarized in Fig. 1.

Flow diagram illustrating the indications for excluding urine samples. A total of 13 dogs met study inclusion criteria and had paired cystocentesis and voided urine samples, each with at least 300 sequence reads.

Of these 19 dogs, mixed breeds were most common (n = 7), followed by Labrador Retrievers (n = 3). The following breeds were each represented once: Beagle, Boxer, Cavalier King Charles Spaniel, Doberman Pinscher, English Bulldog, German Shepherd Dog, Miniature Goldendoodle, Portuguese Water Dog, and Standard Poodle. Mean age of dogs was 3.6 years (± 1.7), and mean weight was 20.6 kg (± 10.5). BCS on a 1–9 scale ranged from 4.5 to 6.5 (median 5.5). Eight dogs had hair length classified as short and 11 as medium. No dogs had long hair. Mean urine pH was 7.3 (± 0.08), and USG ranged from 1.006 to 1.045 (median 1.042). No dogs had glucosuria or ketonuria observed on urinalysis, though 12 dogs exhibited microscopic hematuria (≤ 50 red blood cells/hpf for all but 1 dog). Two urine samples showed scant bacterial growth on aerobic culture (< 10^5^ CFU/mL), and the remaining samples exhibited no observable bacterial growth. Twelve dogs (63%) voided immediately after cystocentesis, which was documented as 0.1 h. The time between cystocentesis and free catch collection ranged from 0.1 to 6 h (median 0.1 h).

Three dogs had an owner-reported history of suspected atopic dermatitis, but the disease was well-controlled with no pruritis or visible dermatologic lesions at the time of sample collection. A separate dog had mild erythema observed on the medial thighs and a history of well-controlled hypothyroidism, but no reported history of dermatologic disease or dermatologic lesions noted in the perivulvar or inguinal regions. All dogs consumed diets commercially formulated to meet the nutritional needs of adult dogs. Thirteen diets were represented across the 19 dogs, with four diets consumed by more than one dog and nine diets consumed by a single participant.

### Filtering and initial data analysis

**Fig. 2:**
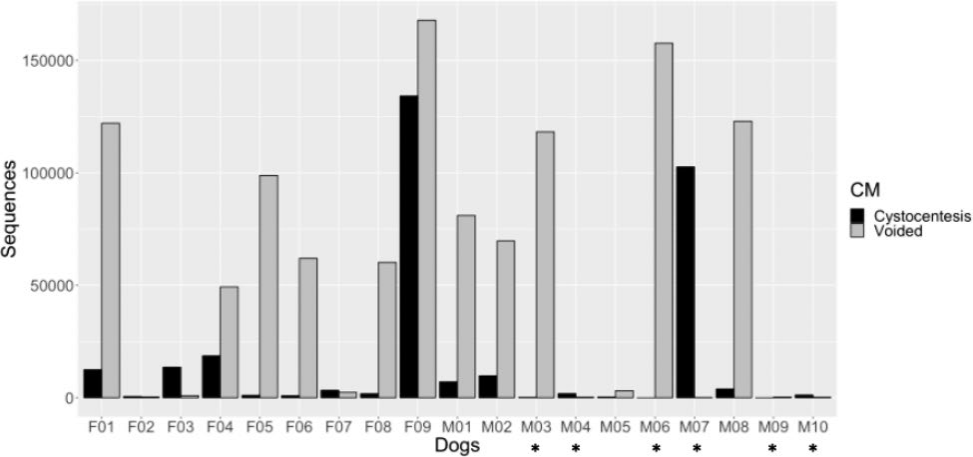
Sequence read counts for paired cystocentesis and voided urine samples from 19 dogs

After initial filtering, eight organisms were identified as contaminants and removed from the dataset prior to downstream analysis (**Additional file 1**). Sequence reads across all samples ranged from 7 to 165,728 reads per sample (median 3649), comprising 414 unique ASVs. Samples collected via cystocentesis had a median of 1850 sequence reads, whereas samples collected via midstream voiding had a median of 61,947 sequence reads (*P* = .036). The voided sample exhibited a higher read count than the paired cystocentesis sample for 13 of 19 dogs. Sequence read counts from each urine sample, reported by dog and collection method, are displayed in Fig. 2.

Sequence reads are reported for each urine sample, female dogs (F01 through F09) and male dogs (M01 through M10). Black bars represent cystocentesis samples, and gray bars represent midstream voided urine. Six dogs were excluded due to insufficient sequence reads (< 300) in at least one sample and are designated with an asterisk (*).

After filtration and putative contaminant removal, no reads were present in either negative control. In the positive control, each of the eight designated mock community members were identified, and no additional organisms were detected. Lowest assigned taxonomy of the mock community organisms was at the family level for *Salmonella enterica*, species level for *Lactobacillus fermentum*, and the remaining organisms were reported at the genus level. The abundance of organisms specified in the commercial product as compared to the abundance of organisms identified from 16 S rRNA gene amplicon sequencing is reported in **Additional file 2**.

Six of 38 urine samples (3 cystocentesis, 3 voided) had less than 300 sequence reads and were removed prior to downstream analysis (Figs. 1 and 2). All were from male dogs, and each sample with insufficient reads was from a different dog. Therefore, 6 dogs were removed for insufficient sequence reads in at least one sample, leaving 13 dogs (4 males, 9 females) that possessed paired samples meeting all study criteria.

### Bacterial composition of urine samples

**Fig. 3:**
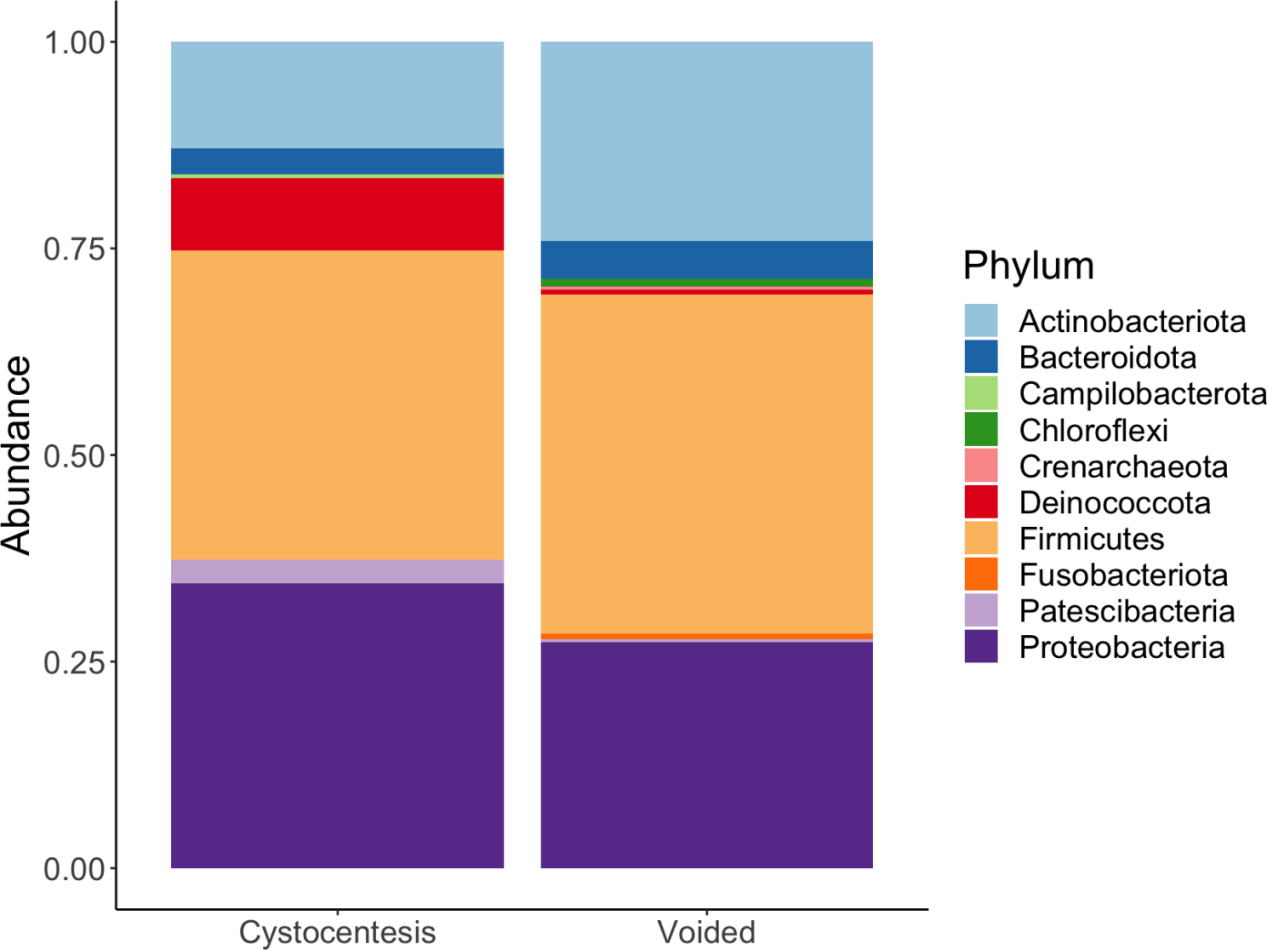
Bacterial phyla present in paired cystocentesis and voided urine samples from 13 dogs

Ten bacterial phyla were represented across all urine samples. Firmicutes, Proteobacteria, and Actinobacteriota were the most common phyla observed across samples regardless of collection method, though relative abundance of specific phyla differed between groups (Fig. 3).

Ten bacterial phyla were represented across samples. Urine collected via cystocentesis exhibited slightly higher proportions of Proteobacteria and Deinococcota and slightly lower proportions of Actinobacteriota as compared to voided urine.

**Fig. 4:**
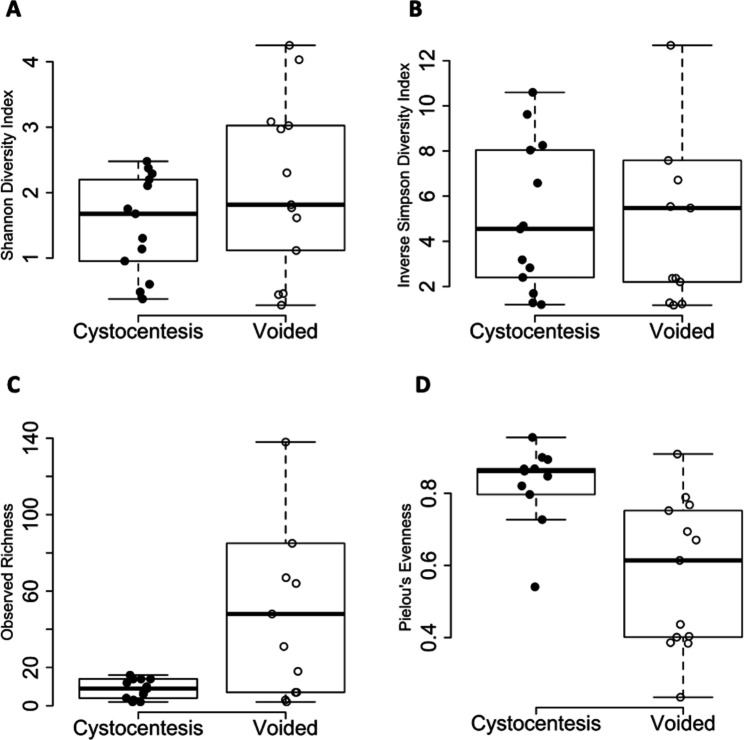
Alpha diversity of paired cystocentesis and voided urine samples from 13 dogs

Differences in alpha diversity were not detected between urine collected via cystocentesis and voided urine based on Shannon diversity index (*P* = .11, Fig. 4A) and inverse Simpson diversity index (*P* = .79, Fig. 4B). However, observed richness was higher in urine collected via midstream voiding than cystocentesis (*P* = .0024; Fig. 4C), whereas Pielou’s evenness was higher in urine collected via cystocentesis than midstream voiding (*P* = .011; Fig. 4D). Voided urine samples exhibited 333 unique ASVs (80% of total ASVs), meaning 333 ASVs were identified in at least one voided sample but in no samples collected via cystocentesis. However, only 10 ASVs (2% of total ASVs) were unique to cystocentesis urine samples and not present in voided urine.

Cystocentesis samples are represented by closed circles, and voided samples are represented by open circles. Boxplot of alpha diversity as measured by the (A) Shannon diversity index (*P =* .11), (B) inverse Simpson diversity index (*P =* .79), (C) observed richness (*P =* .0024), and (D) Pielou’s evenness (*P* = .011). The boxes represent the 25th and 75th percentiles, and whiskers represent 1.5 times the interquartile range.

**Fig. 5:**
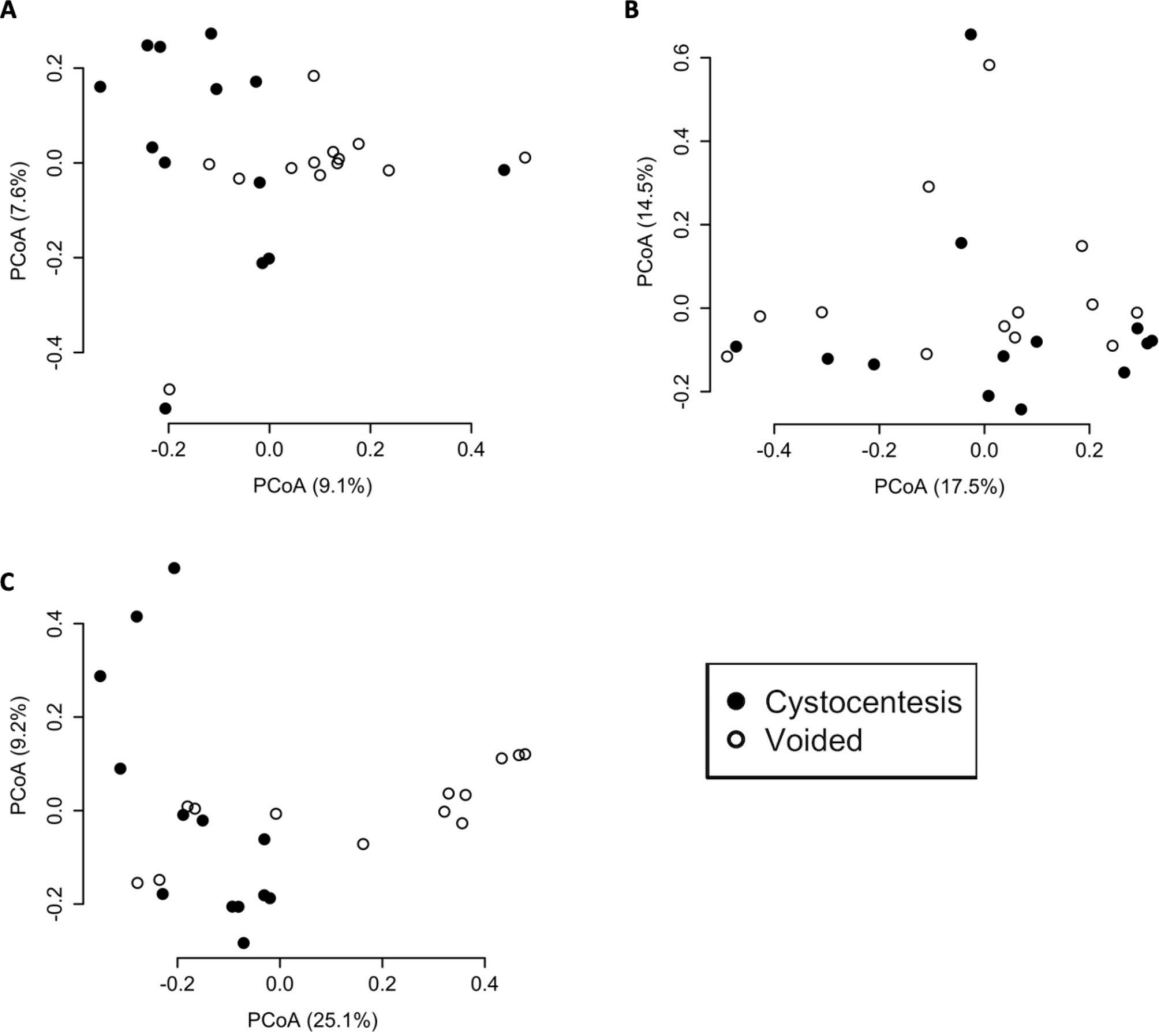
Beta diversity of paired cystocentesis and voided urine samples from 13 dogs

Beta diversity was assessed using three metrics: Bray Curtis dissimilarity matrix (Fig. 5A), Weighted UniFrac (Fig. 5B), and Unweighted UniFrac (Fig. 5C), and tested via PERMANOVA. Both Bray Curtis and Unweighted UniFrac metrics revealed minor but statistically significant differences in overall bacterial composition between cystocentesis and voided urine (*P =* .0050, R^2^ = 0.06 and *P =* .010, R^2^ = 0.07, respectively). Weighted UniFrac did not reveal a difference by collection method (*P* = .31, R^2^ = 0.04). No association was detected between the time interval between urine collections (Spearman’s correlation; *rho* = -0.17, *P =* .60) or hair length classification (*P* = .66) with Bray Curtis dissimilarity for an individual dog’s urine samples.

Principal coordinates analysis (PCoA) plot of beta diversity as measured by (A) Bray Curtis dissimilarity matrix (*P =* .0050, R^2^ = 0.06), (B) Weighted UniFrac (*P* = .31, R^2^ = 0.04), and (C) Unweighted UniFrac (*P =* .010, R^2^ = 0.07). The proportion of variation explained by each axis is listed in parentheses.

No measures of alpha diversity differed between male and female dogs (Shannon diversity index *P =* .72; inverse Simpson diversity index *P* = .89; observed richness *P =* .62; Pielou’s evenness *P* = .96). Beta diversity measures also did not differ by sex (Bray Curtis *P =* .75, R^2^ = 0.04; Weighted UniFrac *P =* .99, R^2^ = 0.02; Unweighted UniFrac *P =* .66, R^2^ = 0.04).

In the two-way PERMANOVA based on Bray Curtis dissimilarities, UM differed by collection method (*P* = .0060). However, there was no observed difference in UM by sex (*P =* .69), nor was there an interaction between sex and collection method (*P* = .53).

### Differential abundance testing

**Fig. 6:**
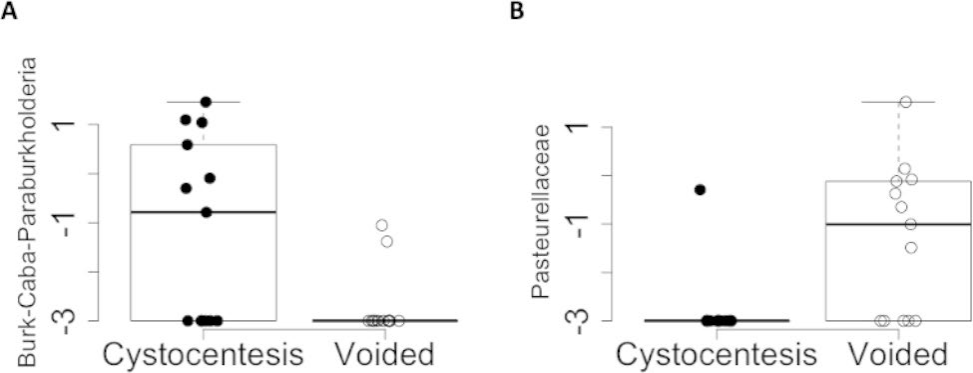
Top differentially abundant taxa between paired cystocentesis and voided urine samples from 13 dogs

Differential abundance of specific taxa between collection method group was assessed using indicator species analysis. Seven ASVs were identified (Table 1) with the criteria of IV > 0.3 and *P* < .05. Two of the differentially abundant ASVs exhibited IVs > 0.5: *Burkholderia-Caballeronia-Paraburkholderia*, which was over-represented in cystocentesis samples, and Pasteurellaceae, which characterized voided samples (Fig. 6). Note that this is a separate *Burkholderia-Caballeronia-Paraburkholderia* ASV than what was previously identified as a contaminant and removed from the dataset.

**Table 1:** Differentially abundant organisms between urine collected by cystocentesis and midstream voiding in 13 dogs

Organism	Group Overrepresented	Indicator Value	*P* Value (Indicator Value)	*P* Value (Wilcoxon signed-rank tests)
Burkholderia-Caballeronia-Paraburkholderia*	Cystocentesis	0.54	0.010	0.021
Pasteurellaceae*	Voided	0.61	0.0070	0.014
Haemophilus*	Voided	0.46	0.012	0.036
*Friedmanniella*	Voided	0.39	0.043	0.059
Streptococcus.2*	Voided	0.039	0.043	0.059
*Streptococcus suis*	Voided	0.039	0.032	0.059
*Fusobacterium*	Voided	0.039	0.039	0.059

Reported are amplicon sequence variants (ASVs) identified by indicator species analysis. Only features with an indicator value (IV) > 0.3 and *P* < .05 for the IV were considered significant. *P* values are also reported for Wilcoxon signed-rank tests, which were used to compare abundance between groups. ASVs with an asterisk (*) represent organisms that were also identified as having high discriminatory power between experimental groups using random forest analysis.

Two amplicon sequence variants with differential abundance between cystocentesis and voided urine samples and an indicator value (IV) > 0.5 are visualized here. Cystocentesis samples are represented by closed circles, and midstream voided samples are represented by open circles. A pseudo-count of 0.01% relative abundance was added to each feature to allow log transformation visualization. Log transformation of relative abundance is displayed in boxplots for (A) *Burkholderia-Caballeronia-Paraburkholderia* (IV = 0.54, *P* for IV = 0.010, Wilcoxon signed-rank test *P =* .021), and (B) Pasteurellaceae (IV = 0.61, *P* for IV = 0.0070, Wilcoxon signed-rank test *P =* .014). The boxes represent the 25th and 75th percentiles, and whiskers represent 1.5 times the interquartile range.

**Fig. 7:**
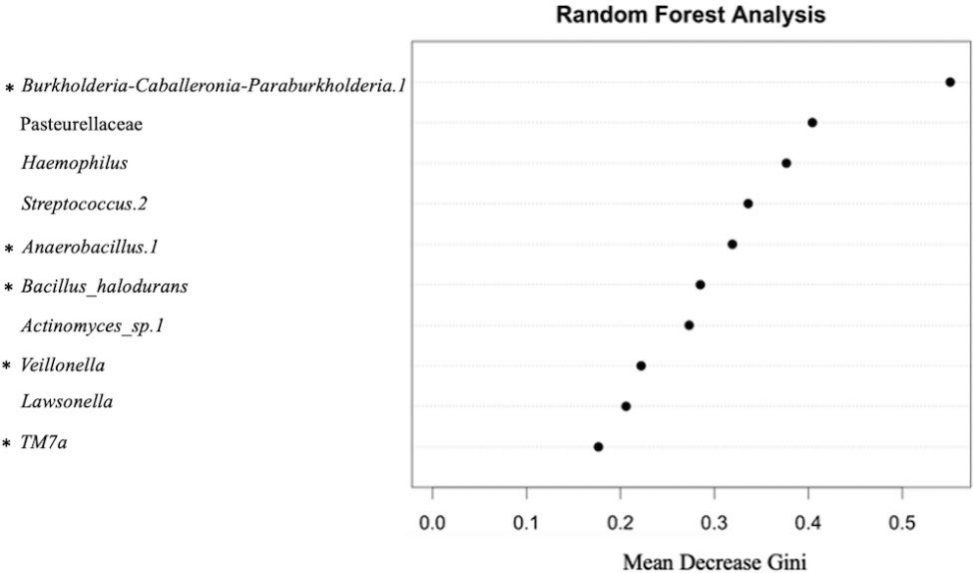
Random forest analysis to detect discriminatory features when classifying canine urine as cystocentesis or voided

Random forest analysis was performed to identify organisms with the highest discriminatory power when classifying samples by collection method (Fig. 7). The top 4 features identified via random forest analysis were also confirmed via indicator species analysis as differentially abundant between collection method groups (Table 1; Fig. 7).

Random forest analysis using classification mode and mean decrease gini index was performed. The top 10 amplicon sequence variants with the highest discriminatory power between cystocentesis and voided urine samples are listed. The top four of these ten features were also identified as differentially abundant between collection technique groups using indicator species analysis. Taxa marked with an asterisk (*) indicate overrepresentation in the cystocentesis group designation. Taxa without an asterisk indicate overrepresentation in the voided group.

No organisms were identified as differentially abundant between males and females when evaluating all urine samples or when looking specifically at samples collected by cystocentesis. However, two organisms significantly differed between males and females when evaluating only voided urine samples. *Schaalia canis* was over-represented in voided urine from female dogs (IV = 0.56; *P* for IV = 0.039, Wilcoxon rank-sum *P =* .029), and *Haemophilus* was over-represented in voided urine from male dogs (IV = 0.69; *P* for IV = 0.031, Wilcoxon rank-sum *P =* .070).

### Comparison of results using different sequence read thresholds and normalization methods

Urine samples and total dogs included with each described sequence read threshold (100, 300, 700, 1000, and 2000 sequence reads) are summarized in **Additional file 3**. Samples meeting the designated threshold ranged from 36 to 38 (95%) at the inclusion criterion of 100 reads to 22 of 38 (58%) at the inclusion criterion of 2000 reads. **Additional file 4** shows a rarefaction curve indicating ASV richness at each sequence read threshold.

The findings from alpha and beta diversity testing between urine collection methods from analyses performed at each sequence read threshold and with each normalization method are summarized in **Additional file 5**. As compared to the primary analysis that used a minimum sequence read requirement of 300 and normalization via relative abundance transformation, patterns of alpha and beta diversity remained largely consistent across analyses despite different read requirements and normalization methods. For alpha diversity measures, differences in observed richness were detected between collection methods at all sequence read thresholds and normalization methods, and Shannon and inverse Simpson indices of alpha diversity were not found to differ by collection method for any sequence read thresholds, except 1000 reads. Pielou’s evenness differed by collection method using relative abundance and DESeq2 normalization for 3 of the 5 sequence read thresholds, including the primary analysis, but never for rarefaction analyses. For beta diversity measures, differences in Bray Curtis and Unweighted UniFrac were detected between collection methods in most (29 of 30) analyses. Maximum R^2^ values for Bray Curtis and Unweighted UniFrac across all analyses were 0.10 and 0.19, respectively, indicating that collection method explained less than 20% of UM variation. Weighted UniFrac did not identify statistically significant differences across any analysis. Finally, no differences in any measure of alpha or beta diversity were detected between male and female dogs using any sequence read cut-off or normalization method (**Additional file 6**).

## Discussion

Our findings demonstrate that the canine UM differs in urine collected by cystocentesis as compared to urine collected by midstream voiding. Voided urine samples possessed significantly higher sequence reads and observed richness than urine collected via cystocentesis, and overall microbial composition differed between collection method based on two of three beta diversity measures. Several ASVs were identified as differentially abundant by collection method, most due to increased abundance in voided samples. No difference in measures of UM diversity were observed between male and female dogs in this study, though two organisms significantly differed by sex when evaluating only voided urine. When measures of UM diversity were compared using different minimum inclusion thresholds for sequence read counts and data normalization methods, overall significance patterns of alpha and beta diversity remained consistent. These findings confirm the presence of diverse microbial communities in canine urine and provide evidence that urine collection technique alters detection of UM composition in dogs.

In the current study, voided samples exhibited significantly higher sequence read counts as compared to urine collected by cystocentesis. While raw sequence read counts are not a direct reflection of sample biomass, we also observed that voided urine harbored roughly 30 times the number of unique organisms and exhibited increased observed richness when compared to cystocentesis samples. These findings likely reflect the presence of microbes from sources other than the bladder, such as the urethra, genital tract, or skin. In humans, there are distinctions in the UM between urine collection method, with voided urine samples often displaying increased abundance of urethral, genital, or peri-urethral/dermal microbes relative to urine collected via more direct bladder sampling (catheterization, cystocentesis, or cystoscopy) [1, 10, 13, 14, 16]. While we did not include microbiota analysis of urethral, genital, or dermal swabs to confirm the origin of specific microbes, all of the differentially abundant organisms over-represented in voided samples (Pasteurellaceae, *Haemophilus, Friedmanniella*, 2 variants of *Streptococcus*, and *Fusobacterium)*, are components of the vaginal or skin microbiota of dogs, supporting the theory that dermal or urethro-genital contamination accounts for the increased abundance of these organisms in voided urine [22, 43–48]. *Fusobacterium* is also an abundant inhabitant of the canine gut microbiota [49, 50]. Taxa from the Pasteurellaceae family have been isolated from pharyngeal swabs in dogs [51], and Pasteurellaceae, *Friedmanniella*, and *Streptococcus* are also known components of the canine oral microbiota [52, 53]. Taxonomic resolution at the species or strain level would allow better interpretation of these findings, though one possibility is that oral contamination secondary to grooming or other behaviors accounts for the over-abundance of these microbes in voided urine.

The biological significance of the single taxon, *Burkholderia-Caballeronia-Paraburkholderia*, over-represented in cystocentesis samples is unclear. This taxonomic group encompasses multiple genera with diverse functions that are associated with both environmental microbes, as well as human and animal pathogens [54]. *Burkholderia* has also been observed in healthy human urine [1, 16]. However, the incomplete taxonomic resolution of this ASV and the identification of a separate *Burkholderia-Caballeronia-Paraburkholderia* ASV as a potential contaminant in this dataset complicate interpretation of this finding.

Urine collected via cystocentesis also exhibited significantly different UM composition compared to voided urine using both Bray Curtis and Unweighted UniFrac measures of beta diversity, but not Weighted UniFrac. This finding suggests that when taking both phylogenetic relatedness and the evenness of organism distribution into account (Weighted UniFrac), voided and cystocentesis samples are more similar. However, the overall presence or absence of organisms was distinct between groups, even when accounting for phylogenetic relatedness (Unweighted UniFrac), and the overall distribution of organisms when not considering phylogenetic relatedness also differed (Bray Curtis). More pronounced changes between urine collection methods via Unweighted UniFrac, as compared to Weighted UniFrac, also occurs in humans when comparing voided to catheterized urine samples [13]. The low R^2^ values for beta diversity by collection method (less than 20% across analyses) indicate that other variables also drive variation between samples. Low R^2^ values have also been observed in human studies evaluating urine collection method [16]. However, the degree of variation that is clinically relevant is unknown, and a low R^2^ might still equate to a meaningful difference.

While we did not detect differences in UM alpha and beta diversity between male and female dogs in this study, the ability to detect differences is likely impacted by study sample size, reproductive status of the dogs, and urine collection technique. For instance, a significant difference in the canine UM was observed in one study using voided samples [24], but not in a separate study that analyzed only cystocentesis samples [22]. In the current study, differential abundance testing within voided urine samples did identify two species that differed by sex: *Haemophilus* (over-represented in males) and *Schaalia canis* (over-represented in females). *Haemophilus* has been well-documented in the male urethra, with various strains of *Haemophilus* shown to associate with urethritis in men [55–58], and *Schaalia canis*, formerly known as *Actinomyces canis* [59, 60], has been previously isolated from the canine vagina [61]. These findings support the notion that urethro-genital microbes can contaminate the UM of voided canine urine samples and contribute to differences between sexes.

The discovery that collection method introduces differences in the UM highlights the need for researchers to employ consistent sample collection methods within a UM study. However, it is important to note that the current study was not designed to evaluate which urine collection technique is superior or more accurately reflects the UM. Rather, the technique should be selected based on the biological question of interest. For instance, a study assessing the UM in dogs with urethral or prostatic disease may warrant the use of voided samples, whereas a study evaluating the UM from dogs with bladder or upper urinary tract disorders may deem cystocentesis or other collection methods most informative. Additionally, researchers should exercise caution when interpreting results across studies that use different urine collection methods.

Towards our secondary objective, we found that patterns of significance for alpha and beta diversity remained largely consistent across analyses using different minimum sequence read thresholds and normalization methods. A rarefaction curve demonstrated that 300 sequence reads, the threshold used for the primary analysis, captured the majority of ASV diversity in most samples. The three methods of data normalization produced only minor differences in *P* values or R^2^ values. The overall consistency between results using various read thresholds and normalization methods offers confidence when interpreting the major findings of this study. However, this study was not designed to evaluate the accuracy or relative superiority of specific normalization techniques, and we direct readers to other resources for more in-depth discussions of data normalization methods, considerations for their use, and their overall performance when evaluating microbial ecosystems [42, 62, 63].

This study has several limitations to consider. First, this population included a small sample size, with a total of only 19 dogs included overall (38 urine samples), but only 13 dogs (26 urine samples) included in the primary analysis after filtering for those with greater than 300 reads present in both paired samples. Additionally, only spayed or neutered dogs were included to mitigate potential confounding variables related to hormonal status. In women, both menopausal-status and menstruation impact UM composition [64, 65]. To date, no studies have evaluated the impact of spay or neuter status on the canine UM; future studies incorporating the impact of collection method on sexually intact dogs is warranted. Another limitation of the study was UM characterization by amplicon sequencing of the bacterial 16 S rRNA gene. While this technique is efficient for taxonomically characterizing microbial communities, this method cannot determine the viability of detected organisms, offers limited taxonomic resolution beyond the genus level, and does not assess the functional potential of organisms. Expanded quantitative urine culture would provide more insight into the viability of these microbial populations [3], and shotgun metagenomics are needed to better define the organisms and their functions, though the low biomass of urine samples poses a challenge with this technique [66]. Finally, only cystocentesis and voided urine were evaluated in the present study; larger prospective studies that also include urethral catheterization or cystoscopic-collected urine could be performed to expand upon these findings.

## Conclusion

Canine urine collected via cystocentesis harbors different microbial communities than urine collected via midstream voiding from the same dog. Contaminant microbes from other anatomic sites likely contribute to the greater abundance of unique microbial organisms in voided urine, supporting the current consensus that the cystocentesis samples better reflect bladder urine. Results were consistent across different analytical methods employed. In sum, urine collection technique affects the composition of urinary microbial communities in dogs. When possible, sample collection methods should be standardized when investigating the canine UM, and when not possible, collection technique should be considered a covariate in the analyses.

## Electronic supplementary material

Below is the link to the electronic supplementary material.

Supplementary Material 1

Supplementary Material 2

Supplementary Material 3

Supplementary Material 4

Supplementary Material 5

Supplementary Material 6

## Data Availability

Raw sequence data generated by and analyzed within this study are available through the National Center for Biotechnology Information (NCBI) Sequence Read Archive (SRA) under the BioProject number PRJNA859856. Other data are included in the manuscript or additional files.

## Abbreviations

ASV: Amplicon sequence variant
BCS: Body condition score
IV: Indicator value
PCoA: Principal coordinates analysis
PERMANOVA: Permutational multivariate analysis of variance
UM: Urinary microbiota
USG: Urine specific gravity
UUF: Unweighted UniFrac
WUF: Weighted UniFrac
