# Supplementary material for: The impact of urine collection method on canine urinary microbiota detection: a cross-sectional study
Source: BMC Microbiol. 2023 Apr 13;23:101. doi: 10.1186/s12866-023-02815-y (PMC10100081; doi:10.1186/s12866-023-02815-y)
Supplement: Supplementary file 1 — Supplementary Material 1 [file 12866_2023_2815_MOESM1_ESM.pdf]

Table S1. Amplicon sequence variants classified as a contaminant via *decontam* in canine urine samples

**Taxonomic resolution**	**Organism**	**P**
Genus	*Escherichia-Shigella*	.17
Genus	*Ruminococcus gnavus group*	.06
Genus	*Paracoccus.1*	.18
Genus	*Anaerobacillus*	.19
Genus	*Paracoccus.2*	.17
Genus	*Cutibacterium*	.14
Genus	*Burkholderia-Caballeronia-Paraburkholderia*	.08
Family	Rhodobacteraceae.1	.04

P refers to a composite score statistic, which is assigned to each feature and classifies taxa as contaminants based on a predetermined threshold value. For the prevalence method with a threshold of P = .5, features are classified as contaminants if identified in a higher fraction of negative controls than in samples [35]. Each amplicon sequence variant identified as a putative contaminant was removed from the dataset prior to downstream analysis.
