# Supplementary material for: The impact of urine collection method on canine urinary microbiota detection: a cross-sectional study
Source: BMC Microbiol. 2023 Apr 13;23:101. doi: 10.1186/s12866-023-02815-y (PMC10100081; doi:10.1186/s12866-023-02815-y)
Supplement: Supplementary file 2 — Supplementary Material 2 [file 12866_2023_2815_MOESM2_ESM.pdf]

Table S2. Summary of the control mock community and respective amplicon sequence variants identified by DNA sequencing

**Mock Community**	**Identification (lowest taxonomic resolution)**	**Mock Community (% Relative Abundance)**	**After sequencing (Raw sequence counts)**	**After sequencing (% Relative Abundance)**
Pseudomonas aeruginosa	*Pseudomonas* (genus)	4.2	11375	14.4
Escherichia coli	*Escherichia-Shigella* (genus)	10.1	9622	12.2
Salmonella enterica	Enterobacteriaceae (family)	10.4	10748	13.6
Lactobacillus fermentum	*Lactobacillus fermentum* (species)	18.4	20347	25.8
Enterococccus faecalis	*Enterococcus* (genus)	9.9	1542	1.95
Staphylococcus aureus	*Staphylococcus* (genus)	15.5	8521	10.8

Listeria monocytogenes	*Listeria* (genus)	14.1	2410	3.1
Bacillus subtilis	*Bacillus* (genus)	17.4	14370	18.2

Eight organisms were included in the commercial mock community used as a positive control. The taxonomic identity and lowest level of taxonomic resolution correlating to each organism are listed. We also report the relative abundance of each organism specified in the product, as compared to the raw sequence counts and relative abundance of each organism from our data after DNA isolation and 16S rRNA gene amplicon sequencing.
