# Supplementary material for: The impact of urine collection method on canine urinary microbiota detection: a cross-sectional study
Source: BMC Microbiol. 2023 Apr 13;23:101. doi: 10.1186/s12866-023-02815-y (PMC10100081; doi:10.1186/s12866-023-02815-y)
Supplement: Supplementary file 3 — Supplementary Material 3 [file 12866_2023_2815_MOESM3_ESM.pdf]

Table S3. Summary of urine samples and dogs meeting minimum sequence thresholds ranging from 100 to 2000.

	**100 reads**	***300 reads**	**700 reads**	**1000 reads**	**2000 reads**
Cystocentesis	17/19	16/19	14/19	13/19	9/19
Voided	19/19	16/19	14/19	13/19	13/19
**Total samples**	**36/38, 95%**	**32/38, 84%**	**28/38, 74%**	**26/38, 68%**	**22/38, 58%**
Males	8/10	4/10	3/10	3/10	3/10
Females	9/9	9/9	8/9	6/9	4/9
**Total dogs**	**17/19, 89%**	**13/19, 68%**	**11/19, 58%**	**9/19, 47%**	**7/19, 37%**

Analyses were performed at five pre-designated thresholds for minimum sequence read counts. For each threshold, the number of urine samples (cystocentesis, voided, and total) and the number of dogs (males, females, and total) meeting the threshold are reported. Dogs were required to have paired urine samples satisfying the sequence read requirement to be included in the counts for those passing the threshold. The primary analysis is designated by an asterisk (*).
