# Supplementary material for: The impact of urine collection method on canine urinary microbiota detection: a cross-sectional study
Source: BMC Microbiol. 2023 Apr 13;23:101. doi: 10.1186/s12866-023-02815-y (PMC10100081; doi:10.1186/s12866-023-02815-y)
Supplement: Supplementary file 4 — Supplementary Material 4 [file 12866_2023_2815_MOESM4_ESM.pdf]

Figure S1. Rarefaction curve of cystocentesis and voided urine samples from 19 dogs

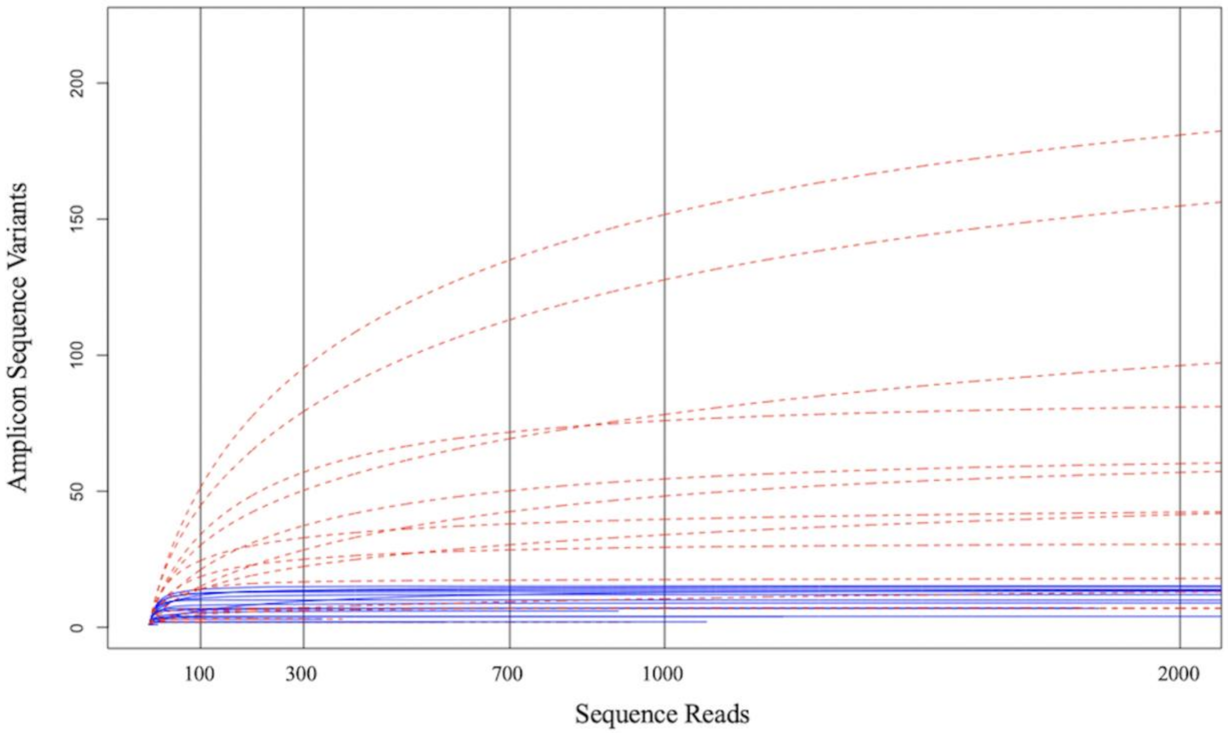

A rarefaction curve shows amplicon sequence variant richness at each designated sequence read threshold (100, 300, 700, 1000, and 2000). Each threshold is indicated by a vertical black line. Red, dashed lines represent midstream voided urine samples. Blue, solid lines indicate cystocentesis urine samples. All 19 original participants are included.
