# Supplementary material for: The impact of urine collection method on canine urinary microbiota detection: a cross-sectional study
Source: BMC Microbiol. 2023 Apr 13;23:101. doi: 10.1186/s12866-023-02815-y (PMC10100081; doi:10.1186/s12866-023-02815-y)
Supplement: Supplementary file 5 — Supplementary Material 5 [file 12866_2023_2815_MOESM5_ESM.pdf]

Table S4. Alpha and beta diversity by collection method using different minimum read thresholds and normalization methods

**Sequence Read Threshold**	**Normalization Method**	**Shannon**	**Inverse Simpson**	**Observed Richness**	**Pielou's Evenness**	**Bray Curtis (R^2)**	**WUF (R^2)**	**UUF (R^2)**
100 reads	Rarefaction	.49	.72	**.04**	.17	**.01 (.04)**	.59 (.03)	**.01 (.04)**
	Relative Abundance	.24	.93	**.006**	**.013**	**.007 (.04)**	.40 (.03)	**.002 (.07)**
	DESeq2	.24	.93	**.006**	**.013**	**.01 (.04)**	.55 (.03)	**.003 (.07)**
*300 reads	Rarefaction	.19	.49	**.01**	.094	**.01 (.05)**	.10 (.06)	**.003 (.09)**
	*Relative Abundance	.11	.79	**.002**	**.011**	**.005 (.06)**	.31 (.04)	**.01 (.07)**
	DESeq2	.11	.79	**.002**	**.011**	**.02 (.06)**	.25 (.05)	**.001 (.11)**
700 reads	Rarefaction	.07	.46	**.01**	.12	**.002 (.07)**	.07 (.07)	**.001 (.14)**
	Relative Abundance	.10	.64	**.005**	**.032**	**.006 (.07)**	.24 (.06)	**.003 (.13)**
	DESeq2	.10	.64	**.005**	**.032**	**.008 (.07)**	.16 (.06)	**.001 (.14)**

1000 reads	Rarefaction	**.02**	.57	**.004**	.13	**.008 (.08)**	.19 (.08)	**.001 (.17)**
	Relative Abundance	**.04**	.65	**.004**	.055	**.008 (.09)**	.13 (.08)	**.002 (.15)**
	DESeq2	**.04**	.65	**.004**	.055	**.016 (.08)**	.06 (.09)	**.001 (.16)**
2000 reads	Rarefaction	.08	.94	**.016**	.11	**.04 (.10)**	.10 (.11)	**.003 (.19)**
	Relative Abundance	.11	.94	**.016**	.11	**.03 (.10)**	.06 (.12)	**.003 (.16)**
	DESeq2	.11	.94	**.016**	.11	.07 (.10)	.15 (.11)	**.003 (.17)**

P values for Wilcoxon signed-rank test and for PERMANOVA are reported to demonstrate statistical significance of alpha and beta diversity measures between urine collected by cystocentesis versus midstream voiding. R^2 values are listed in parentheses for beta diversity measures. *P* values < .05 are in bold. Each analysis is designated by a number of reads, which represents the minimum sequence read threshold that samples must possess to be included in that analysis. The number is followed by rarefaction, relative abundance, or DESeq2 to indicate which normalization method was used. The primary analysis is designated by an asterisk (*). WUF = Weighted UniFrac; UUF = Unweighted UniFrac.
